# Construction of Recombinant Human GM-CSF and GM-CSF-ApoA-I Fusion Protein and Evaluation of Their Biological Activity

**DOI:** 10.3390/ph14050459

**Published:** 2021-05-13

**Authors:** Mariya Pykhtina, Svetlana Miroshnichenko, Vladimir Romanov, Antonina Grazhdantseva, Galina Kochneva, Anatoly Beklemishev

**Affiliations:** 1 Institute of Biochemistry of Federal Research Center of Fundamental and Translational Medicine (FRC FTM), Novosibirsk, 2 Timakova Street, Novosibirsk 630117, Russia; Pykhtina_maria@mail.ru (M.P.); svmiro@yandex.ru (S.M.); vprom@mail.ru (V.R.); 2 Federal Budgetary Research Institution State Research Center of Virology and Biotechnology “Vector”, Koltsovo, Novosibirsk 630559, Russia; gaa@vector.nsc.ru (A.G.); g.v.kochneva@yandex.ru (G.K.)

**Keywords:** expression in *Pichia pastoris*, ryGM-CSF, ryGM-CSF-ApoA-I fusion protein, TF-1 cells, bone marrow cells (BMC), survival, ApoA-I

## Abstract

In this study, two strains of the yeast *P. pastoris* were constructed, one of which produced authentic recombinant human granulocyte-macrophage colony-stimulating factor (ryGM-CSF), and the other was a chimera consisting of ryGM-CSF genetically fused with mature human apolipoprotein A-I (ApoA-I) (ryGM-CSF-ApoA-I). Both forms of the cytokine were secreted into the culture medium. The proteins’ yield during cultivation in flasks was 100 and 60 mg/L for ryGM-CSF and ryGM-CSF-ApoA-I, respectively. Both forms of recombinant GM-CSF stimulated the proliferation of human TF-1 erythroleukemia cells; however, the amount of chimera required was 10-fold that of authentic GM-CSF to induce a similar proliferative effect. RyGM-CSF exhibited a 2-fold proliferative effect on BFU-E (burst-forming units—erythroid) at a concentration 1.7 fold less than non-glycosylated *E. coli*-derived GM-CSF. The chimera together with authentic ryGM-CSF increased the number of both erythroid precursors and BMC granulocytes after 48 h of incubation of human bone marrow cells (BMCs). In addition, the chimeric form of ryGM-CSF was more effective at increasing the viability of the total amount of BMCs, decreasing apoptosis compared to the authentic form. ryGM-CSF-ApoA-I normalized the proliferation, maturation, and segmentation of neutrophils within the physiological norm, preserving the pool of blast cells under conditions of impaired granulopoiesis. The chimera form of GM-CSF exhibited the properties of a multilinear growth factor, modulating the activity of GM-CSF and, perhaps, it may be more suitable for the normalization of granulopoiesis.

## 1. Introduction

GM-CSF is a pleiotropic cytokine that plays a key role in the proliferation and differentiation of many hematopoietic cells, especially monocytes, granulocytes, and dendritic cells [1]. In clinical practice, recombinant GM-CSF preparations are used in combination with chemotherapy and radiotherapy in the treatment of cancer patients [2] or in bone marrow transplantation in order to reduce the risk of infections [3]. In addition, the possibility of using GM-CSF in the treatment of neurodegenerative diseases [4,5,6], immunotherapy of malignant diseases [7,8], and in autoimmune conditions [9,10] to accelerate wound healing [11,12,13] has recently been investigated.

GM-CSF is synthesized by a variety of cells, including lymphocytes, fibroblasts, endothelial cells, monocytes, and some malignant cells [14]. Human GM-CSF consists of 127 amino acids and has two potential N-glycosylation sites and several O-glycosylation sites [15]. The heterogeneity of glycosylation results in a wide variability in the apparent molecular weight of GM-CSF, ranging from 14.5 to 32 kDa [16]. The degree of glycosylation of GM-CSF can influence its biological activity, pharmacokinetics, immunogenicity, and toxicity [17,18,19].

Currently, there are two recombinant GM-CSF preparations on the pharmaceutical market: Sargramostim and Molgramostim, which contain recombinant GM-CSF produced by *Saccharomyces cerevisiae* yeast and *Escherichia coli* bacteria, respectively. Molgramostim contains non-glycosylated GM-CSF with an additional N-terminal methionine. Its use is accompanied by autoimmune reactions to this cytokine and a high incidence of side effects, as a result of which the drug was not approved by the FDA for use in therapy in the United States [20,21]. Sargramostim contains mature glycosylated GM-CSF and is FDA approved for clinical use. However, the disadvantage of using *S. cerevisiae* yeast as a producer of therapeutic proteins is that they produce hyperglycosylated proteins, in which each oligosaccharide can contain more than 50 mannose residues, as a result of which the biological activity of these proteins is reduced [22].

The methylotrophic yeast *Pichia pastoris* has several advantages for the production of therapeutic proteins: namely, they carry out post-translational protein modifications similar to those in humans; do not produce exo- and endotoxins; provide a high yield of recombinant proteins; and their secretion into the culture medium greatly facilitates the purification of recombinant protein [23].

Due to the severe side effects associated with its use, GM-CSF is used in the clinic much less frequently than G-CSF. One of the side effects of GM-CSF is the escalation of inflammation [24]. In addition, the half-life of GM-CSF, similar to most other recombinant therapeutic proteins, is very short and requires frequent administration to maintain effective concentrations [25].

To increase the clinical efficacy and reduce the side effects of recombinant GM-CSF, researchers use two main strategies for its modification: covalent binding to PEG (pegylation) [26,27] and the creation of genetically engineered fusion proteins. Pegylation often leads to the appearance of an inhomogeneous difficult-to-clean mixture of products, a low yield of cytokines, and a decrease in its biological activity [28]. In addition, PEG can induce the formation of anti-PEG antibodies [29,30,31].

The fusion construct is a chimeric molecule containing rGM-CSF fused to a recombinant protein that often serves two functions. First, it has beneficial properties and in a functional sense complements the cytokine; secondly, it ensures protection of the cytokine from the action of proteases and thus prolongs the activity of the cytokine in the human body.

Currently, there are many developments of this type of construction that can be widely used in clinical medicine: GM-CSF fused to IL-3 is used to stimulate hematopoiesis [32]; GM-CSF fused to Bcl-XL as an anti-apoptotic therapeutic agent [33]. Members of a new class of immunomodulatory cytokines, fusokines, which are pairs of two cytokines fused together, e.g., GM-CSF fused to IL-2, IL-15, or IL-21, are used for immunotherapy [34]; GM-CSF fused to blood plasma transferrin is suitable for the treatment of neurodegenerative diseases [35] and fused to albumin is suitable for the immunotherapy of tuberculosis [36].

In this study, we have obtained recombinant human GM-CSF fused to human ApoA-I. The choice of ApoA-I was not accidental, since this protein performs many functions in the body, such as antiatherogenic [37], antiapoptotic [38], antithrombotic [39], and antioxidant [40]. In addition, it has been shown that a decrease in the level of ApoA-I in the body is associated with the development of a number of pathological conditions: its low level is associated with the development of a number of oncological diseases [41]; in patients with sepsis, a decreased ApoA-I level is associated with a poor prognosis of survival [42]. Additionally, recent data show that a decrease in serum ApoA-I levels is a prognostic sign for the development and severity of COVID-19 [43,44].

This protein is involved in various anti-inflammatory strategies of the body, suppressing the TLR-mediated secretion of the pro-inflammatory cytokines IL-6 and TNF in monocytes [45]. By affecting the production of IL-1beta [46], ApoA-I may be useful in the treatment of infectious diseases, including COVID-19, which is manifested by increased levels of IL-6, TNF, and IL-1beta [47]. ApoA-I also has several valuable carrier protein properties: namely, it circulates in the body for a long time, is readily biodegradable, is not immunogenic, and binds specifically to most cell types due to the presence of SR-BI receptors [48].

Therefore, the production of a recombinant GM-CSF-ApoA-I chimera might mitigate the toxic effect of GM-CSF on target cells, both due to the anti-inflammatory properties of ApoA-I in the chimera and due to the expected more prolonged action of the chimera in the blood, consequently decreasing its dose. Additionally, it is not known whether the biological activity of the cytokine will be fully preserved. In the literature, there are examples of a decrease in the biological activity of cytokines in the development of fusion hybrid constructs [49,50,51]. Parameters such as the type, length, and flexibility of the linker, as well as the way the cytokine binds to the protein (at the C or N-terminus of the module) can directly affect the biological activity of the fusion protein.

In connection with the previous research, it was of interest to compare the biological activities of recombinant authentic and chimeric forms of human GM-CSF. In this work, both forms of recombinant human GM-CSF produced by *P. pastoris* X33 strains were obtained. The biological activities of authentic and chimeric GM-CSF forms were studied using the human TF-1 erythroid line and human bone marrow cells.

## 2. Results

### 2.1. Creation of the Recombinant P. pastoris Strain Capable of Producing Authentic and Chimeric Forms of Human GM-CSF

#### 2.1.1. Creation of Recombinant Plasmids Encoding an Authentic and Chimeric Form of Mature Human GM-CSF

At the first stage of our work, we constructed recombinant plasmids encoding an authentic and chimeric form of mature human GM-CSF. The synthetic gene of mature human GM-CSF optimized for expression in *P. pastoris* cells and designated as ryGM-CSF was synthesized by DNA2.0 (USA) and cloned into plasmid pD912 at the XhoI and SalI sites. We used the plasmid pD912/ryGM-CSF as the source of the ryGM-CSF gene to construct the recombinant plasmids pPICZα-A-rhGM-CSF and pPICZα-A/rhGM-CSF-link-ApoA-I.

The recombinant plasmid pD912/ryGM-CSF was digested with restriction endonucleases either XhoI and SalI or XhoI and KpnI. The excised ryGM-CSF gene was inserted into the pPICZαA plasmid at the XhoI and SalI sites and cloned into *E. coli* Top10 cells.

**Figure 1:**
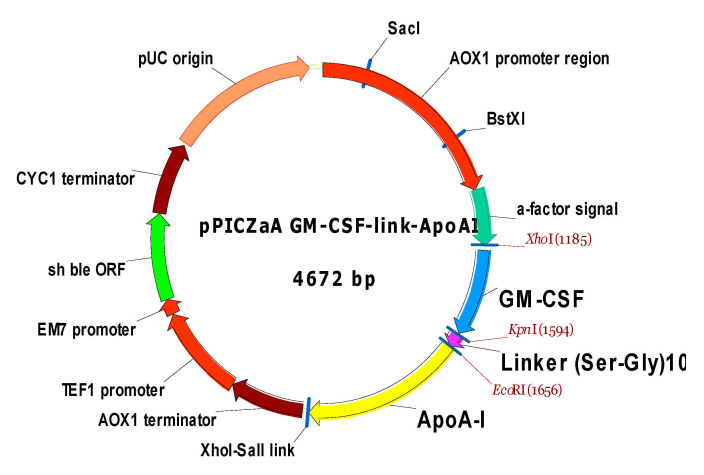
Construction of the pPICZαA/ryGM-CSF-link-ApoA-I recombinant plasmid.

In parallel, the previously constructed plasmid pPICZαA/IFN-link-ApoA-I was cleaved at the XhoI and KpnI sites, and thus, the IFN gene was excised from it. The ryGM-CSF gene was inserted into the cleaved plasmid at the XhoI and KpnI sites, and the resulting recombinant plasmid was transformed into *E. coli* Top10 competent cells. The scheme of the constructed recombinant plasmid pPICZαA/ryGM-CSF-link-ApoA-I is shown in Figure 1.

**Figure 2:**
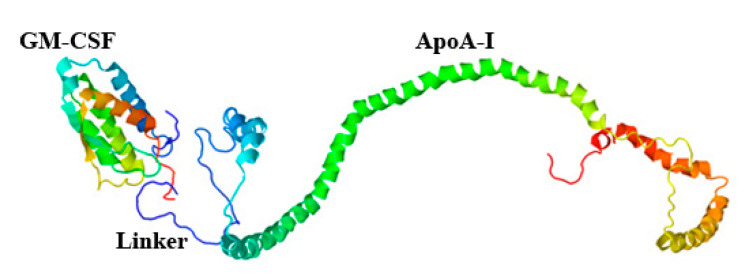
Structural 3D model of ryGM-CSF-ApoA-I.

Plasmid pPICZαA/ryGM-CSF-link-ApoA-I was designed to express the chimeric protein ryGM-CSF-ApoA-I. To determine if the ryGM-CSF function would be potentially impaired due to a possible change in the conformation of cytokines in the chimera, a hypothetical 3D model was built using the RaptorX protein structure prediction server (http://raptorx.uchicago.edu/, accessed on 6 May 2021). As seen in Figure 2, the three-dimensional structures of ryGM-CSF and ApoA-I are spatially separated from each other, and thus, the function of both proteins in the chimera can be expected to be retained.

*E. coli* clones bearing the target recombinant plasmids were selected both on selective LB agar medium containing 50 μg/mL zeocin and by PCR of colonies with primers AOX1-F and AOX1-R used for the detection and sequencing of genes cloned into the pPICZαA plasmid. The sizes of the amplicons were determined by electrophoresis in 0.8% agarose gel stained with ethidium bromide. Selected clones containing pPICZαA/ryGM-CSF or pPICZαA/ryGM-CSF-link-ApoA-I plasmids were used for their preparative isolation and subsequent cloning in *P. pastoris* X33 cells.

#### 2.1.2. Transformation of *P. pastoris* Cells with Recombinant Plasmids and Screening of Transformants

Competent cells of the yeast *P. pastoris* X33 were transformed with plasmids pPICZαA/ryGM-CSF and pPICZαA/ryGM-CSF-link-ApoA-I and grown on YPD agar plates containing 2000 μg/mL zeocin. On day 4, about 10 colonies were grown on plates containing yeast cells transformed with plasmid pPICZαA/ryGM-CSF and about 25 colonies on plates containing cells transformed with pPICZαA/ryGM-CSF-link-ApoA-I. Then, zeocin-resistant transformants were evaluated for their ability to synthesize and secrete the recombinant cytokines ryGM-CSF or ryGM-CSF-ApoA-I. Clones producing the largest amount of proteins with a molecular weight close to the molecular weight of the target proteins ryGM-CSF and ryGM-CSF-ApoA-I were used for subsequent studies.

**Figure 3:**
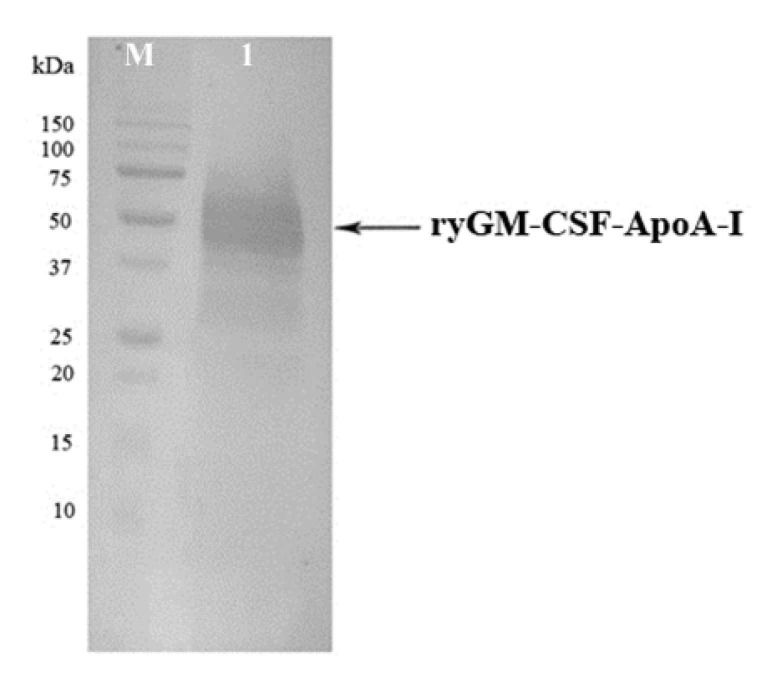
Western blotting of proteins secreted by *P. pastoris* clone No 3 into culture medium. Lanes: (**M**) the pre-stained standard molecular weight marker (10–150 kDa) (BioRad); (**1**) ryGM-CSF-ApoA-I expressed in *P. pastoris*.

The results of one assay of yeast clones to produce of the chimeric cytokine ryGM-CSF-ApoA-I are shown in Figure S1. All clones selected on a medium containing 2000 μg/mL of zeocin produced and secreted different amounts of chimeric cytokine. The presence of ApoA-I in the ryGM-CSF-ApoA-I chimera was confirmed by Western blotting using rabbit anti-ApoA-I IgG (Figure 3).

Clones No # 7 and No 3, producing the highest amounts of ryGM-CSF and ryGM-CSF-ApoA-I, respectively, were selected for preparative production and purification of these cytokines. Notably, according to reports [52,53], the introduction of non-ionic detergents into the culture medium increases the yield of the secreted protein and prevents their aggregation. We tested whether the addition of 0.2% (*w*/*v*) non-ionic detergent Tween 20 to the culture medium would affect the expression efficiency of both authentic and chimeric ryGM-CSF in our case. The addition of 0.2% (*w*/*v*) Tween 20 increased the yield of ryGM-CSF and ryGM-CSF-ApoA-I by 2.5–3 times (figures not shown). In this regard, the preparative production of recombinant cytokines was carried out by culturing the corresponding strains in the presence of 0.2% (*w*/*v*) Tween 20.

#### 2.1.3. Cultivation of *P. pastoris* Strains Producing ryGM-CSF and ryGM-CSF-ApoA-I, and Purification of Recombinant Cytokines

**Figure 4:**
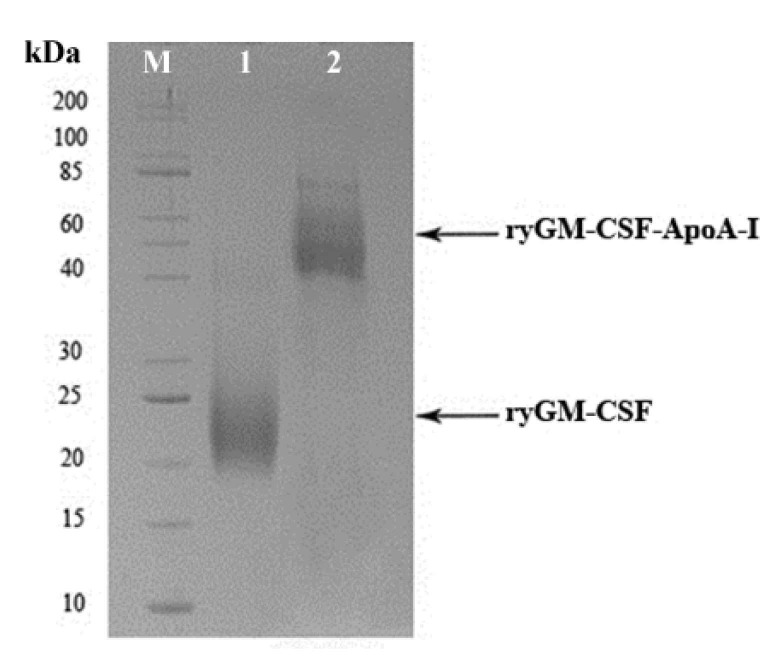
SDS-PAGE analysis of ryGM-CSF and ryGM-CSF-ApoA-I preparations purified by successive ion exchange chromatography steps. Lanes: (**M**) standard molecular weight marker (10–200 kDa) (Sib Enzyme); (**1**) sample of ryGM-CSF; (**2**) sample of ryGM-CSF-ApoA-I.

Selected yeast clones producing the highest amounts of ryGM-CSF and ryGM-CSF-ApoA-I were grown in conical flasks with deflectors in BMGY medium on an orbital shaker at 25 °C and 250 rpm. During cultivation in the presence of the inducer, daily aliquots of the culture liquid were sampled and analyzed by electrophoresis in SDS-PAGE to assess the dynamics of synthesis and secretion of recombinant cytokines (Figure S2). At the end of induction, yeast cells were removed by centrifugation, and proteins from supernatants were precipitated by the addition of dry ammonium sulfate to 75% saturation at 4 °C overnight. Before the addition of ammonium sulfate, aliquots of the supernatants were taken to assess the quantitative yield of cytokines. We found that the total yield of ryGM-CSF and ryGM-CSF-ApoA-I at the end of cultivation was approximately 100 mg/L and 60 mg/L, respectively. Protein precipitates obtained after precipitation with ammonium sulfate were dissolved, dialyzed, and used for chromatographic purification of both forms of ryGM-CSF. Dialysate containing ryGM-CSF was purified by successive chromatography on DEAE-Sepharose columns (at pH 4.5 and 7.5) and SP Sepharose FF. The dialysate containing ryGM-CSF-ApoA-I was purified by two successive chromatography cycles on DEAE-Sepharose columns (chromatography at pH 4.5) and on a SP Sepharose FF column. Aliquots of the eluted fractions were analyzed by electrophoresis on 12% PAGE. The fractions containing the highest amount of the purified cytokines were pooled and stored in 100 μL aliquots at −70 °C until use. The final preparations of the chromatographically purified both forms of cytokines were analyzed by electrophoresis in 12% SDS-PAGE (Figure 4). Both authentic and chimeric ryGM-CSF were approximately 95% pure by electrophoretic analysis. Purified preparations of both ryGM-CSF forms were used to study their biological activity.

### 2.2. Biological Activity of Recombinant Yeast GM-CSF and GM-CSF-ApoA-I

#### 2.2.1. Erythroid Stimulating Activity of ryGM-CSF and ryGM-CSF-ApoA-I on Erythroleukemia Cells TF-1

The erythroid-stimulating activity of ryGM-CSF and ryGM-CSF-ApoA-I was evaluated in human TF-1 erythroleukemia cells. GM-CSF more strongly supports the proliferation and viability of myeloid progenitors due to the direct proliferative stimulus on all progenitors and precursors of the granulomonopoietic lineage. GM-CSF is also a powerful stimulator of burst forming unit-erythroid (BFU-E) and megakaryocyte colony forming unit (CFU-MK) proliferation.

**Figure 5:**
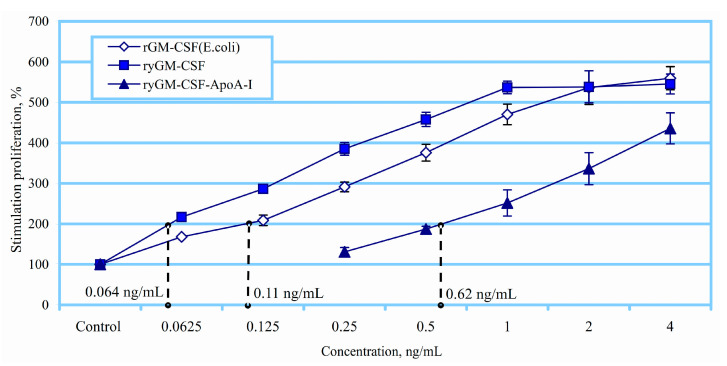
Influence of ryGM-CSF and ryGM-CSF-ApoA-I on the proliferation of TF-1 cells. Data are presented as mean ± standard error (SEM).

In this regard, the biological activity of recombinant yeast GM-CSF (ryGM-CSF) was assessed using a culture of human erythroleukemia cells TF-1, which is GM-CSF-dependent and proliferates in the presence of GM-CSF and other human cytokines. rGM-CSF, which was expressed in *E. coli* cells (GM-CSF), was used as a reference protein for quantitative evaluation [54]. The experiments were carried out over a wide range of concentrations of the calibration protein. Figure 5 shows the correlation of proliferative activity of the ryGM-CSF and standard rGM-CSF. As follows from the experimental results, a 2-fold proliferative effect on TF-1 cells was achieved at a concentration of the control rGM-CSF—of 0.11 ng/mL, while to achieve a similar effect, the ryGM-CSF produced in yeast cells required only 0.064 ng/mL (Figure 5).

Thus, ryGM-CSF is a more potent stimulator of the proliferation of erythroid explosive units (BFU-E) compared to the control rGM-CSF, exhibiting 1.7-fold greater biological activity against TF-1 cells. At the same time, ryGM-CSF-ApoA-I activated the proliferation of TF-1 cells, but it exhibited less activity compared to rGM-CSF and to ryGM-CSF bacterial preparation and to yeast GM-CSF. A 2-fold proliferative effect on TF-1 cells was achieved at a concentration of 0.62 ng/mL.

#### 2.2.2. Myeloid-Stimulating Activity of ryGM-CSF and ryGM-CSF-ApoA-I on Human Bone Marrow Cell

##### Analysis of BMC Using Flow Cytometry

Recombinant GM-CSF is most widely used in the prevention of neutropenia and neutropenic complications in patients with a reduced number of neutrophils in the blood. In this regard, the bone marrow of a person with a reduced level of neutrophils and impaired maturation was taken as a sample for the study of the recombinant growth factors.

**Figure 6:**
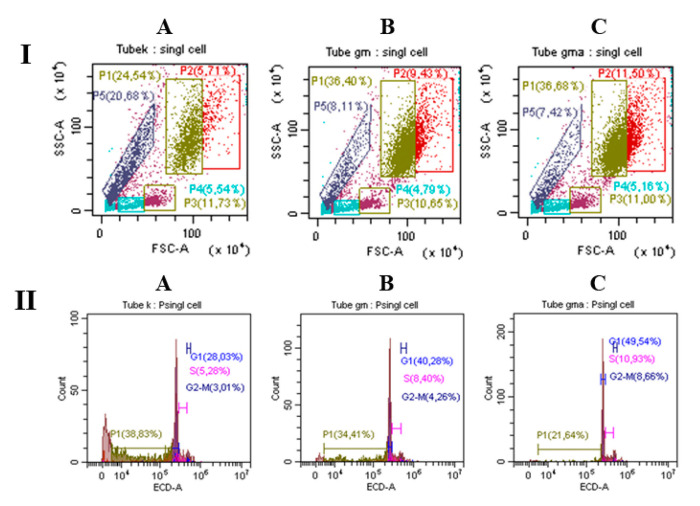
Flow cytometric analysis (**I**) and cell cycle of all BMCs (**II**) treated of ryGM-CSF (**C**) and ryGM-CSF-ApoA-I (**B**) compared to the control after 48 h of incubation (**A**).

BMC were incubated with growth factors for 48 h. The effect of ryGM-CSF and ryGM-CSF-ApoA-I was analyzed by flow cytometry (Figure 6). Under the influence of both growth factors, the total number of viable BMC cells increased after 48 h of incubation (this difference was especially pronounced in the case of ryGM-CSF-ApoA-I).

In percentage terms, the largest difference in the granulocyte lineage of BMC was in the case of ryGM-CSF (36.4% maturing cells, 9.4% dividing cells) and ryGM-CSF-ApoA-I (36.7% maturing cells, /11.5% dividing cells) (Figure 6I). Analysis of the cell cycle showed that in the presence of the chimera, the number of cells in the active cycle was higher ((S + G_2_M phases) −19%) compared to ryGM-CSF (12%) and the control (8%) (Figure 6II).

Additionally, the percentage of apoptotic cells decreased to 21% under the influence of ryGM-CSF-ApoA-I, to 34% in the case of GM-CSF, and to 38% in the control after 48 h of incubation. A more significant decrease in apoptosis and an increase in the level of proliferation in the presence of ryGM-CSF-ApoA-I provides an increase in the total number of viable cells in comparison with both the authentic form and the control (1340 ± 167 in control samples; 1885 ± 75 and 2055 ± 61 for GM-CSF and ryGM-CSF-ApoA-I, respectively).

Notably, in this case, the additive effect of ApoA-I and GM-CSF was likely manifested. ApoA-I is known to increase cell viability, although in these cases, it was used in microgram amounts. During our research, both factors were used in nanogram amounts. This underlines that both recombinant proteins exhibit the properties of growth factors.

##### Myelography

For a more complete understanding of the effect of growth factors, human BMC smears were prepared and stained and the number of cells was counted: 500 per glass.

Analysis of stained smears of human BMC samples showed that in the control, after 24 h of incubation, the number of cells of the granulocyte line was reduced and amounted to 27% of the total BMC population.

The general decrease in the number of cells of the granulocytic series in the control was accompanied by an increase in the number of cells of the monocytic series: monocytes, macrophages and stromal cells. Both factors increased the number of granulocyte cells significantly (to 51 ± 6%) (data taken from complete myelogram). Notably, the control samples also contained an increased number of blast forms of granulocyte cells (8.8 ± 0.3%) and an increased number of hyposegmented neutrophils (26 ± 0.35%). 

**Figure 7:**
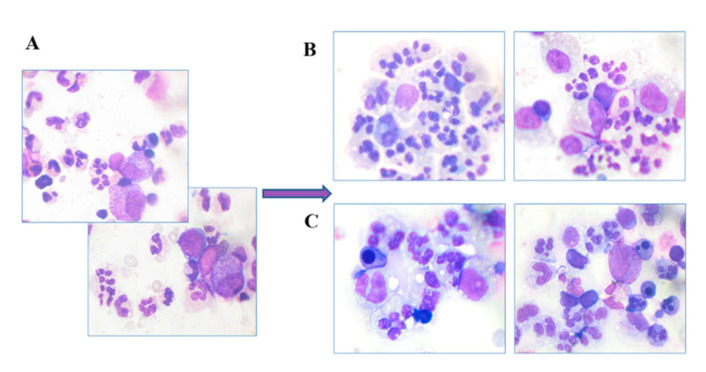
Human BMCs treated with ryGM-CSF-ApoA-I (**B**) and ryGM-CSF (**C**) compared to a control (**A**) for 24 h of incubation. (**A**) Blast cells, a small number of segmented neutrophils, stable neutrophils; (**B**,**C**) many segmented neutrophils.

Growth factors significantly increased the number of normally segmented neutrophils, reducing the percentage of cells with abnormal segmentation after 24 h of incubation (Figure 7).

**Figure 8:**
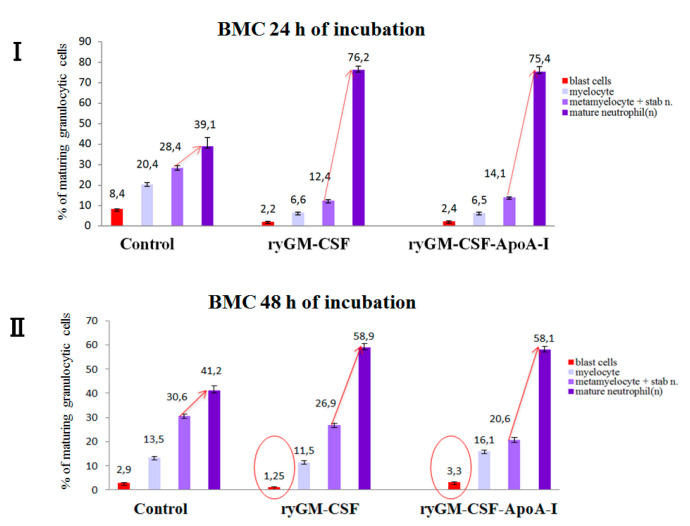
Dynamics of changes in cells content of the granulocytic series under the influence of recombinant ryGM-CSF and ryGM-CSF-ApoA-I during after 24 (**I**)–48 (**II**) hours of incubation ((**I**): all cell groups under growth factors statistically differ from the control groups, values are mean ± SD (*n* = 4), *p* < 0.01; (**II)**: statistically significant differences between blast cells treated with ryGM-CSF and ryGM-CSF-ApoA-I, *p* < 0.01).

To visualize the dynamics of changes (24–48 h) in the cellular composition of granulocyte cells in the experimental and control samples, the cells were divided into groups:

(a) blast forms of granulocytic cells (myeloblasts, promyelocytes); (b) young forms (myelocytes—maternal and daughter); (c) maturing cells (metamyelocytes, stabe neutrophils); (d) mature forms of granulocytes (segmented neutrophils) (Figure 8).

As can be seen from Figure 8, in the control samples, the maturation of neutrophils was visibly impaired, since the number of stabe neutrophils and metamyelocytes is practically equal to the number of segmented neutrophils during incubation at both 24 and 48 h. Growth factors significantly stimulate the normal maturation of neutrophils, doubling the number of segmented neutrophils within 24 h of incubation. In the process of maturation, the number of neutrophil abnormalities and blast cells decreased (Figure 7).

The dynamics of cell maturation in control samples at 48 h of incubation did not change compared to 24 h. In the control at 48 h of incubation, a significant reduction in all forms of blast cells, including granulocytic cells and an increase in the level of apoptosis, was noted.

The data were consistent with the results of flow cytometry, where there was an increased apoptosis, an increase in cell debris, and a low level of proliferation (Figure 6A) of control cells after 48 h of incubation.

In the presence of ryGM-CSF, a significant increase in the number of mature granulocytes after 1 day of incubation was accompanied by a further decrease in the number of blast forms of the granulocyte on the second day of incubation. The chimeric form stimulated the growth of granulocyte cells a similar way during 24 h of incubation while maintaining the number of blast forms on the second day of incubation (increase from 2.5% to 3.2%) (Figure 7).

This agreed with the increased level of human BMC proliferation during incubation (48 h) in the presence of the chimeric form as assessed by flow cytometry (Figure 6B,C). According to myelogram data, both factors activate blast cells of the erythroid lineage; however, the effect of ryGM-CSF was more effective on the first day (3.9% of cells were proerythroblasts), while in the presence of the chimeric form, the number of proerythroblasts was reduced (1.1%).

The number of erythroid blast cells under the influence of both factors became equal and amounted to (6.1 ± 0.3%) after 48 h of incubation. The authentic form demonstrated the full spectrum of multilinear differentiation; there was also a small number of megakaryoblasts. At the same time, these cells were not observed in the presence of a chimeric cytokine. The chimera well supported the viability of the reticular–stromal system of the bone marrow. This effect may have been due to the presence of ApoA-I in the chimera (Figure S3). Therefore, the recombinant cytokines exhibit the properties of growth factors, maintaining the viability of human BMC after 48 h of incubation. Both factors normalized granulocytopoiesis, reducing the number of hyposegmented neutrophils and increasing the total number of cells of the granulocyte series while maintaining the number of blast forms. Furthermore, the proliferative activity of the cells in the presence of the chimera was higher, and the apoptotic death was lower than in the case of ryGM-CSF, which contributed to an increase in the total number of cells. This suggests that the chimeric form may be more effective than ryGM-CSF in the treatment of diseases with the inhibition of myeloid hematopoiesis, which is accompanied by a reduced level of neutrophils and (or) impaired maturation.

## 3. Discussion

Recombinant GM-CSF is used in clinical practice for the treatment of a wide range of hematopoietic diseases, including chemotherapy-induced neutropenia, and it is also being investigated for the treatment of several other diseases (neurodegenerative, autoimmune, cancer, wound healing, etc.). A significant limitation of its use is its high toxicity and short half-life in humans. The toxicity of GM-CSF is associated with the activation of macrophages and the synthesis of pro-inflammatory cytokines.

GM-CSF produced by bacterial cells is also highly toxic and immunogenic due to the presence of additional N-terminal methionine and impurities of LPS-producing cells, which are very difficult to purify. One of the possible approaches to solving the problem of GM-CSF toxicity is the creation of its potentially prolonged form by fusion with other proteins.

We have obtained authentic GM-CSF as well as its chimeric form by expression in the *P. pastoris* methylotrophic yeast. One of the main advantages of heterologous gene expression in these microorganisms over bacterial expression systems is the inherent ability of methylotrophic yeast to secrete synthesized heterologous proteins and glycosylate them in much the same way as in humans [55].

Glycosylated rhGM-CSF has a better pharmacokinetic profile than bacterial (non-glycosylated) rhGM-CSF, which is characterized by a relatively stable plasma concentration [18]. Stable cytokine concentration may be therapeutically more effective than the high peaks and sharp reduction of non-glycosylated rhGM-CSF with relatively short half-life.

We have selected human apolipoprotein A-I as the fusion protein. A flexible oligopeptide linker was provided between the pair of fusion proteins, which in our case made it possible to preserve the activity inherent in GM-CSF, with modulation of its properties by ApoA-I. Notably, the creation of chimeric proteins is often accompanied by a significant decrease in the biological activity of the cytokine, despite the presence of linkers in the chimera.

Preparative production of recombinant proteins was carried out on a thermostatic shaker in flasks. The protein yield during cultivation was about 100 and 60 mg/L for ryGM-CSF and ryGM-CSF-ApoA-I, respectively. Considering that protein production took place under non-optimized conditions, it can be expected that fermentation in the bioreactor would significantly increase the yield of recombinant proteins.

The study of the biological activity of the obtained cytokines was carried out both on a standard human erythroleukemia cell line TF-1 and on human bone marrow cells. Hematopoietic growth factor GM-CSF, which ensures the proliferation of several hematopoietic germs, is also a potent stimulator of erythroid proliferation (BFU-E activity), but it does not affect more mature erythroid cells [56]. Our study showed that both forms of ryGM-CSF support the viability of early progenitor cells of the erythroid lineage. The efficiency of ryGM-CSF produced by *P. pastoris*, which was studied on cytokine-dependent cells of human erythroleukemia TF-1, was higher than that of the chimeric form of ryGM-CSF-ApoA-I and the recombinant GM-CSF synthesized in *E. coli.*

Analysis of the human BMC myelogram showed four times more proerythroblasts in samples stimulated for 24 h with ryGM-CSF compared to samples stimulated with ryGM-CSF-ApoA-I. However, on the second day, the number of erythroid progenitors was equal in both BMC samples. Transformed TF-1 cells have an unlimited number of divisions, while in the bone marrow, there are fewer progenitors and more different factors to maintain their viability and proliferation are required. Under these conditions, the chimeric cytokine was sufficient to soft activate erythroid progenitors within 48 h, while authentic ryGM-CSF exhausted this potential within 24 h. Slower activation of erythroid units in the presence of a chimera can be explained by steric hindrances of its interaction with receptors on erythroid cells.

Considering that ApoA-I itself, used in microgram amounts, stimulates the proliferation of granulo/monocytic progenitor cells and maintains their viability [57], it can be assumed that ApoA-I fused with GM-CSF has an additive effect on the survival and proliferation of these cells.

Notably, the property of ApoA-I to support cell viability and proliferation was manifested in a chimeric form, which was taken in ng quantities, while ApoA-I per se did not show this activity in such a quantity. The property of maintaining proliferation, reducing apoptotic cell death, and preserving the pool of progenitors was most fully manifested at 48 h of incubation. The chimeric form better supported the viability of human BMC, promoting the maturation of cells of the granulocyte series and reducing the number of hyposegmented neutrophils more effectively than in the control or in samples treated with ryGM-CSF. The obtained chimera retained the ability of the authentic factor to support the cells of the erythroid lineage. According to the results obtained, the chimera can be considered as a new multilinear factor with GM-CSF activity, which is supplemented by the ability to maintain cell survival. The property of a chimeric protein to increase cell viability may be especially important for the stimulation of hematopoiesis after chemotherapy and radiotherapy. Cells stimulated to proliferation are highly sensitive to unfavorable environmental factors, and a decrease in apoptotic cell death is essential for therapy with growth-stimulating factors. In this case, the ability of the chimera to increase the viability of proliferating cells in adverse conditions is relevant for possible use in the therapy of myelosuppression of cancer patients.

We have previously obtained two other recombinant human colony stimulation factors produced by *P. pastoris* yeast cells: authentic ryG-CSF and ryG-CSF fused to human ApoA-I (ryG-CSF-ApoA-I). The biological activity of these cytokines was tested on rat and human bone marrow cells. Both ryG-CSF and ryG-CSF-ApoA-I stimulated the proliferation of the granulocytic series cells. In addition, the ryG-CSF-ApoA-I chimera exhibited new functions not characteristic of G-CSF; they supported the viability of the lymphocytic lineage cells, as well as macrophage-stromal cells, which, as was previously established, is characteristic of ApoA-I [57]. As mentioned, ApoA-I itself is a biologically active protein that exhibits many properties in the body. The regulatory effect of ApoA-I on the proliferation and stem properties of mesenchymal stromal cells is shown in Miroshnichenko [58]. However, it should be emphasized that the activity of ApoA-I was manifested at concentrations [57,58] three orders of magnitude higher than the concentration of the recombinant forms of ryGM-CSF-ApoA-I and ryG-CSF-ApoA-I. Other researchers obtained human interferon α2b (IFN) fused with ApoA-I, which showed more pronounced immunostimulatory activity, reduced hematotoxicity, and better pharmacokinetic properties compared to authentic IFN [59].

These data indicate that ApoA-I in the composition of the chimera endows the cytokines fused with it with additional new properties that can be useful for the treatment of a number of diseases. It is possible that the additive effect of the ApoA-I protein and the growth factor GM-CSF in the chimera will manifest itself in models of alveolar macrophages. It has recently been suggested that GM-CSF is required for the clearance of cholesterol from alveolar macrophages, with a decrease in this clearance being a major macrophage defect leading to pulmonary alveolar proteinosis [60]. The importance of ApoA-I for the reverse transport of cholesterol and its anti-inflammatory role in pulmonary disease has been noted in studies [61,62]. There is more and more evidence in the literature indicating the role of ApoA-I in the pathogenesis and severity of lung disease: ApoA-I mimetic peptides have been found to be useful in the treatment of lung diseases such as viral pneumonia [63], pulmonary hypertension [64], asthma [65], and acute respiratory distress syndrome [66].

The biological properties of the obtained ryGM-CSF-ApoA-I have yet to be investigated in more detail. Comparison of the pharmacokinetic profiles of authentic recombinant ryGM-CSF and its chimeric form is of great interest. The presence of the ApoA-I protein and glycosylated ryGM-CSF in the chimera likely provides increased stability of the chimeric protein in plasma.

## 4. Materials and Methods

The non-glycosylated human GM-CSF with a methionine residue at the N-terminus produced in *E. coli* and with bioactivity equivalent to that of commercial rhGM-CSF was kindly provided by Dr. Gileva I.P. Restriction endonucleases were purchased from SibEnzyme (SibEnzyme, Novosibirsk, Russia). T4 DNA ligase and Phusion DNA-polymerase were purchased from Thermo Fisher Scientific Inc. (Thermo Fisher Scientific Inc., Vilnius, Lithuania). Oligonucleotides were purchased from LTD “Biosynthesis” (Biosynthesis, Novosibirsk, Russia). Yeast extract, bacto peptone, and triptone were purchased from Thermo Fisher Scientific Inc. (Thermo Fisher Scientific Inc, Bleiswijk, Netherlands) and were used for preparations of Luria–Bertani (LB) medium for the growing of *E. coli.* Yeast culture media (YPD, BMGY, BMM2, BMM10) were prepared as specified in the manufacturer’s protocol “EasySelectTMPichia Expression Kit” (Invitrogen, Carlsbad, CA, USA). RPMI media, fetal bovine serum (FBS), penicillin, and streptomycin were purchased from Thermo Fisher Scientific Inc. (Thermo Fisher Scientific Inc, Bleiswijk, Netherlands); XTT and Propidium Iodide were purchased from Sigma-Aldrich (Sigma-Aldrich, Saint Louis, MO, USA). Sephadex G25, DEAE-Sepharose FF, and SP Sepharose FF ion-exchange resins were purchased from GE Healthcare Bioscience (GE Healthcare Bioscience, Uppsala, Sweden). PVDF transfer membrane were purchased from Thermo Fisher Scientific Inc, (Thermo Fisher Scientific Inc, Rockford, IL, USA). The water used in this study was deionized and autoclaved.

### 4.1. Bacterial and Yeast Strains, and the Plasmid Vector

*Escherichia coli* str. TOP10, *Pichia pastoris* str. X33, and the pPICZα-A were purchased from Invitrogen Inc. The recombinant pPICZα-A/hIFN-link-ApoA-I plasmid was constructed earlier in our laboratory. *E. coli* str. TOP10 was used for the cloning and propagation of synthetic human GM-CSF in pPICZa-A and pPICZα-A/hIFN-link-ApoA-I plasmids. *Pichia pastoris* str. X33 was used for cloning pPICZa-A/hGM-CSF and pPICZa-A/hGM-CSF-link-ApoA-I plasmids and expression and production of the authentic and chimeric forms of recombinant human GM-CSF.

### 4.2. Creation of the Recombinant P. pastoris Strain Capable of Producing Authentic and Chimeric Forms of Human GM-CSF

#### 4.2.1. Design of a Synthetic Gene Encoding Mature Human GM-CSF

The construction and optimization of the nucleotide sequence encoding the synthetic gene of mature human GM-CSF to ensure its efficient expression in *P. pastoris* was carried out using the Gene Designer software (ATUM, Newark, CA, USA). The synthetic gene designated as ryGM-CSF contained a XhoI restriction site and a nucleotide sequence encoding a Kex2 proteolysis site and two Ste13 proteolysis sites at the 5’-terminus, and a KpnI restriction site, a stop codon, and restriction sites EcoRI and SalI at the 3’-terminus. The optimized gene was synthesized by ATUM and cloned into plasmid pD912 at the XhoI and SalI sites. The synthesized and cloned sequence of the ryGM-CSF gene was confirmed by sequencing. The recombinant plasmid pD912/GM-CSF was used as a synthetic gene source for mature human GM-CSF.

#### 4.2.2. Construction of the pPICZαA/ryGM-CSF Plasmid

The recombinant plasmid pD912/ryGM-CSF was digested with restriction endonucleases XhoI and SalI and analyzed by electrophoresis in 0.8% agarose gel. A fragment containing the ryGM-CSF gene was eluted from the gel and ligated with T4 DNA ligase with the plasmid pPICZαA, which had preliminarily been cleaved at the XhoI and SalI sites.

#### 4.2.3. Construction of pPICZαA/ryGM-CSF- link-ApoA-I Plasmid

The pPICZαA/IFN-link-ApoA-I plasmid, constructed previously in our laboratory earlier, was used as the initial vector. The plasmid contained a chimeric gene containing in the 5’-end region the human interferon-α2b (IFN) gene sequence flanked by the XhoI and KpnI restriction sites and fused to a linker encoding the GSSGSGSSGSGSGSSGGSG sequence. The linker was attached with the mature human ApoA-I gene, flanked by EcoRI and SalI restriction sites and located in the 3’-terminal region of the chimera. The IFN gene was removed from the plasmid by digestion with restriction enzymes XhoI and KpnI and replaced with the synthetic ryGM-CSF gene inserted into the same restriction sites using T4 DNA ligase to form the recombinant plasmid pPICZαA/ryGM-CSF-link-ApoA-I.

#### 4.2.4. Transformation of *E. coli* Cells and Screening of Transformants

*Escherichia coli* str. TOP10 electrocompetent cells were transformed with recombinant plasmid pPICZαA/ryGM-CSF or pPICZαA/ryGM-CSF-link-ApoA-I using the electroporator MicroPulser (Bio-Rad, Hercules, CA, USA) as described previously Aliquots of 50 and 100 µL of LB medium, containing transformed *E. coli* str. TOP10 cells were spread onto low salt Luria–Bertani agar plates comprised of 1% (*w*/*v*) tryptone, 0.5% (*w*/*v*) yeast extract, 0.5% (*w*/*v*) NaCl, 1.8% (*w*/*v*) bactoagar and 50 μg/mL of zeocin. Clones grown on agar medium were checked by colony PCR for the presence of recombinant plasmids containing inserts of the synthetic gene ryGM-CSF and the chimeric gene ryGM-CSF-ApoA-I. PCR was performed using the DNA Thermal Cycler BIS (BIS, Novosibirsk, Russia) in the presence of forward AOX1-F 5’-GACTGGTTCCAATTGACAAGC-3’ and reverse AOX1-R 5’-CAAATGGCATTCTGACATCC-3’ primers used for the detection and sequencing of genes cloned in pPICZαA plasmid. The sizes of the amplicons were determined by electrophoresis in 0.8% agarose gel stained with ethidium bromide. Selected clones containing the pPICZαA/ryGM-CSF or pPICZαA/ryGM-CSF-link-ApoA-I plasmid were used for their preparative isolation and subsequent cloning in *P. pastoris* X33 cells.

#### 4.2.5. Transformation of *P. pastoris* Cells and the Selection of Recombinant Clones

Plasmids pPICZαA/ryGM-CSF and pPICZαA/ryGM-CSF-link-ApoA-I were digested with restriction endonuclease BstXI, and their linearized forms were used to transform electrocompetent *P. pastoris* X33 cells using a MicroPulser electroporator (Bio-Rad, Hercules, CA, USA). Transformants were grown on YPD agar plates containing 2000 μg/mL zeocin in an incubator at 30 °C for 3–5 days. Then, zeocin-resistant transformants were evaluated for their ability to synthesize and secrete the recombinant cytokines ryGM-CSF or ryGM-CSF-ApoA-I by culturing the clones in 96-well plates (2 mL square wells). The cultivation was carried out in BMGY (1% yeast extract, 2% peptone, 100 mM potassium phosphate, pH 6.0, 1.34% YNB, 4 × 10^5^% biotin and 2% glycerol) medium on an orbital shaker at 300 rpm for 60 h at 28°C. Then, 250 μL of BMM2 (same as BMGY with 1% methanol instead of glycerol) was added to each well. Over the next three days, 50 μL BMM10 (same as BMGY with 5% methanol instead of glycerol) was added to the wells.

On the 4th day of induction, cells from the cultures of each well were pelleted by centrifugation. Proteins from selected supernatants were precipitated with 10% TCA and analyzed for the presence of the target protein by SDS-PAGE electrophoresis. Clones producing the largest number of proteins with a molecular weight close to the molecular weight of the target proteins ryGM-CSF and ryGM-CSF-ApoA-I were used for subsequent studies. The antigenic nature of the ryGM-CSF-ApoA-I chimera was determined by Western blotting using rabbit anti-ApoA-I IgG [67].

#### 4.2.6. Growing Yeast Cells Producing Target Proteins

All procedures for growing yeast clones were performed as described previously. Selected clones were grown in 50 mL of BMGY medium for two days on an orbital shaker Unimax 2010 (Heidolph Instruments GmbH & Co, Schwabach, Germany) at 250 rpm and 28 °C. Then, the culture was transferred to 100 mL of BMGY medium in 500 mL conical baffled flask and grown in an orbital shaker at 25 °C and 250 rpm. Then, 0.2% (*w*/*v*) Tween 20 was added to the culture medium, and 1% methanol was added daily On the 4th day after induction, the cells were pelleted by centrifugation; then, ammonium sulfate was added to the supernatant containing the target protein to a concentration of 75% saturation and incubated at 4 °C overnight. Protein precipitates were harvested by centrifugation at 39,000 *g* using a JA20 rotor in a J2–21 centrifuge (Beckman Coulter, Indianapolis, IN, USA) for 30 min at 4 °C.

### 4.3. Chromatographic Purification of Recombinant Cytokines

#### 4.3.1. Purification of ryGM-CSF

The pellets containing mainly ryGM-CSF were dissolved in buffer A, containing 25 mM sodium acetate, 1 mM EDTA, 0.02% Tween 20, pH 4.5, and dialyzed against buffer A. The resulting dialyzed was loaded to DEAE-Sepharose and equilibrated with a buffer A. The fraction unbound to resin (breakthrough) was loaded onto an SP Sepharose FF column equilibrated with the same buffer. The column was washed with buffer A, and the protein was eluted with a NaCl gradient (0–0.5 M). Fractions were analyzed by electrophoresis on 12% PAGE. Fractions containing the highest amount of purified target protein were pooled and dialyzed against buffer B, containing 10 mM sodium phosphate and 1 mM EDTA, (pH 7.5). The dialyzed solution was loaded to a DEAE-Sepharose column equilibrated with buffer B. The column was washed with buffer B and the protein was eluted with a NaCl gradient (0–0.25 M). All fractions were analyzed using SDS-PAGE (12%). The fractions containing the highest amount of the target protein were pooled and stored in 100 μL aliquots at −70 °C until use.

#### 4.3.2. Purification of ryGM-CSF-ApoA-I

The pellet, containing mainly the ryGM-CSF-ApoA-I chimera, was dissolved in buffer A. The solution was desalted on a Sephadex G25 column. The demineralized chimera solution was loaded onto a DEAE-Sepharose column equilibrated with the buffer B. Unbound to resin material was collected and loaded to an SP Sepharose FF column equilibrated with the buffer B. Then, the column was washed with buffer B and the protein was eluted with a NaCl gradient (0–0.5 M). Aliquots of the eluted fractions were analyzed by electrophoresis on 12% PAGE. Fractions containing the highest amount of chimeric protein were pooled and stored in 100 μL aliquots at −70 °C until use.

#### 4.3.3. Determination the Concentration of Recombinant Cytokines

The concentrations of ryGM-CSF and ryGM-CSF-ApoA-I were measured spectrophotometrically by UV absorption at a wavelength of 280 nm, considering their molar extinction coefficients as well as densitometry of cytokine bands stained with Coomassie brilliant blue in a gel after SDS-PAGE versus standard human serum albumin (HSA) titration bands using analytical imaging software Gel-Pro analyzer (Media Cybernetics, Silver Spring, MD, USA).

## 5. Analysis of the Biological Activity of ryGM-CSF and ryGM-CSF-ApoA-I

### 5.1. XTT Assay

The biological activity of ryGM-CSF and ryGM-CSF-ApoA-I was evaluated by the level of stimulation of human erythroleukemia cells TF-1 proliferation using 2,3-Bis-(2-methoxy-4-nitro-5-sulfophenyl)-2H-tetrazolium-5-carboxanilid (XTT) (Sigma-Aldrich). To calibrate the activity of both forms of recombinant human yeast GM-CSF (ryGM-CSF and ryGM-CSF-ApoA-I), a recombinant GM-CSF preparation obtained by expression in *E. coli* cells was used [54]. We prepared 2-fold dilutions of growth factors to their final concentration of 0.063, 0.125, 0.25, 0.5, 1, 2, and 4 ng/mL (calibration) in RPMI medium (Thermo Fisher Scientific Inc.) supplemented with 10% fetal calf serum. A 96-well plate was seeded at the rate of 2 × 10^4^ cells/well in the culture medium with the investigated amounts of the cytokines and incubated for 72 h at 37 °C, 5% CO_2_, and 85% humidity. Cells incubated without added cytokines served as negative controls. The optical density was determined on a SpectraCount plate spectrophotometer (Packard) OP 490/620. The stimulation of proliferation of TF-1 cells was calculated as a percentage relative to the control. The number of living cells in the negative control was taken as 100%. All calculations were performed using the LabView software.

### 5.2. Myeloid Stimulating Activity of ryGM-CSF and ryGM-CSF-ApoA-I on Human Bone Marrow Cell

#### 5.2.1. Cell Culture

In this study, we used human bone marrow cells (BMC) taken from the culture bank of Research Institute of Clinical and Experimental Lymphology—Branch of the ICG SB RAS (RICEL–branch ICG SB RAS). Cells were cultured in cell culture medium RPMI (Thermo Fisher Scientific Inc.) with 10% fetal bovine serum (FBS) under standard cultivation condition (37 °C, 5% CO_2_, 20% O_2_ at humidified atmosphere). BMCs were cultivated in 24-well culture plates at a density of 2 × 10^6^ cells/mL in the presence of the ryGM-CSF or ryGM-CSF-ApoA-I (50 ng/mL) for 24 and 48 h. Cells cultured under the same conditions, but without additives of cytokines, were used as a control.

#### 5.2.2. Flow Cytometry

BMCs were analyzed by flow cytometry (CYTOFLEXS-100, Beckman Coulter, Brea, CA, USA) as described previously [67]. Cells types were gated based on cell size and granularity (FSC/SSC). The cell cycle was estimated using Propidium Iodide (PI) staining. The cells were harvested and fixed in ice-cold 70% ethanol for 2 h, and incubated for 30 min in a hypotonic solution of propidium iodide (Sigma-Aldrich, St. Louis, MO, USA) with RNase Thermo Fisher Scientific Inc. (Bleiswijk, The Netherlands).

#### 5.2.3. Myelography

Bone marrow smears were prepared and stained with May-Grunwald and Giemsa dyes. Stained cells were assessed microscopically (Axio Scope A1 fluorescence microscope (Zeiss, Oberkochen, Germany)), and 500 BMCs per smear were counted.

## 6. Conclusions

*P. pastoris* strains that efficiently produce both authentic and chimeric forms of human GM-CSF were obtained. The chimera ryGM-CSF-ApoA-I exhibited the properties of a growth factor, demonstrating the activity of GM-CSF, increasing the number of cells of the granulocyte lineage on BMC and BFU-E.

At the same time, ryGM-CSF-ApoA-I increased the viability of the total amount of BMC and reduced apoptotic cell death more efficiently than ryGM-CSF. The chimeric form normalized the proliferation, maturation, and segmentation of neutrophils within the physiological norm, preserving the pool of blast cells under conditions of impaired granulopoiesis. These results indicate the modulating effect of ApoA-I in the composition of the chimera on the biological activity of GM-CSF and the advisability of a more in-depth study of the properties of the chimeric cytokine.

## Data Availability

The data presented in this study are available on request from the corresponding author. The data are not publicly available due to privacy restrictions and patient confidentiality.

## Supplementary Materials

The following are available online at https://www.mdpi.com/article/10.3390/ph14050459/s1, Figure S1: SDS-PAGE analysis of TCA-precipitated proteins secreted by P. pastoris clones into culture medium after 96 h of induction. Lane M contains a standard protein molecular weight marker (Sib Enzyme). Lanes1–8 contains TCA-precipitated proteins of analyzed clones, Figure S2: SDS-PAGE analysis of ryG-CMSF-ApoA-I production on the orbital shaker. Lane M: protein molecular weight marker (Sib Enzyme); lanes 1–4 contains 24, 48, 72, 96 h by methanol induction. Samples of culture supernatants that were 15-fold concentrated with TCA. Figure S3: Human BMCs treated with ryGM-CSF–ApoA-I (B) and ryGM-CSF (C) versus control (A) for 48 h of incubation. The control is observed: Immature hyposegmented neutrophils, increased apoptosis, phagocytosis, increased number of monocytes. Samples treated with ryGM-CSF–ApoA-I-mature neutrophils, viable intact stroma, blast cells. Samples treated with ryGM-CSF-mature neutrophils with less condensed chromatin, monocytes, macrophages, eosinophils and erythroid blasts, apoptosis.

## Abbreviations

GM-CSF	Granulocyte-Macrophage Colony-Stimulating Factor
ApoA-I	Apolipoprotein A-I
BMC	Bone Marrow Cells
BFU-E	Burst Forming Unit-Erythroid
CFU-MK	Colony-Forming Unit-Megakaryocyte
SDS-PAGE	Sodium Dodecyl Sulfate–Polyacrylamide Gel Electrophoresis
LPS	Lipopolysaccharides
HAS	Human Serum Albumin
PI	Propidium Iodide
FBS	Fetal Bovine Serum
