# Supplementary material for: Construction of Recombinant Human GM-CSF and GM-CSF-ApoA-I Fusion Protein and Evaluation of Their Biological Activity
Source: Pharmaceuticals (Basel). 2021 May 13;14(5):459. doi: 10.3390/ph14050459 (PMC8152757; doi:10.3390/ph14050459)
Supplement: Supplementary file 1 [file pharmaceuticals-14-00459-s001.zip › pharmaceuticals-1192106-supplementary.pdf]

pharmaceuticals

Supplementary Materials:

Construction of recombinant human GM-CSF and GM-CSF-ApoA-I fusion protein and assessment of their biological activity.

Mariya Pykhtina[1], Svetlana Miroshnichenko[1], Vladimir Romanov[1], Antonina Grazhdantseva[2], Galina Kochneva[2] and Anatoly Beklemishev[1],*

[1] Institute of Biochemistry of Federal Research Center of Fundamental and Translational Medicine (FRC FTM), Novosibirsk, 2 Timakova Street, 630630 Novosibirsk, Russia;
M.P.: Pykhtina_maria@mail.ru; S.M.: svmiro@yandex.ru; V.R.: vprom@mail.ru;

[2] Federal Budgetary Research Institution State Research Center of Virology and Biotechnology "Vector", Koltsovo, Novosibirsk region, Russia
A.G.: gaa@vector.nsc.ru; G.K.: g.v.kochneva@yandex.ru

* Correspondence: beklem@niibch.ru (A.B.)

Supplementary Materials:

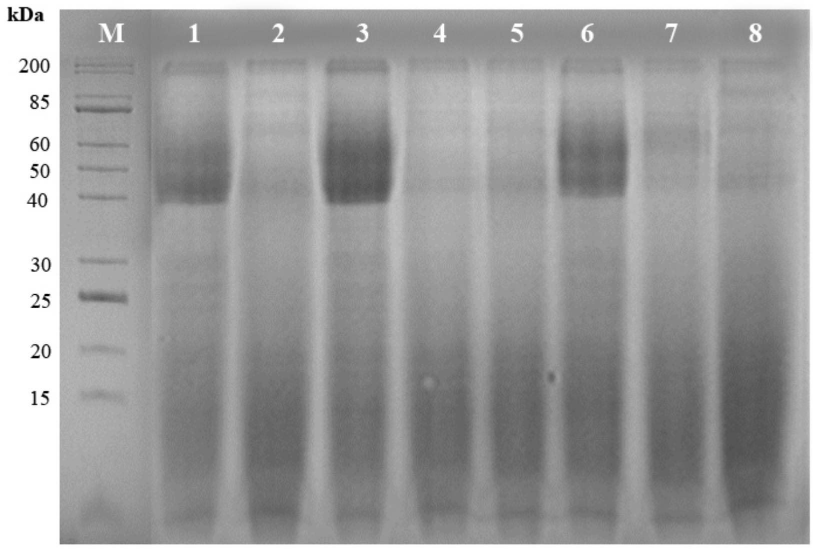

Figure S1. SDS-PAGE analysis of TCA-precipitated proteins secreted by P. pastoris clones into culture medium after 96 h of induction. Lane M contains a standard protein molecular weight marker (Sib Enzyme). Lanes1–8 contains TCA-precipitated proteins of analyzed clones.

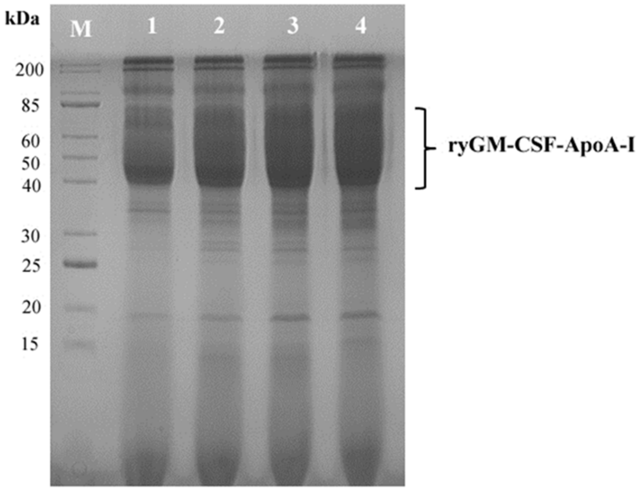

Figure S2. SDS-PAGE analysis of ryG-CMSF-ApoA-I production on the orbital shaker. Lane M: protein molecular weight marker (Sib Enzyme); lanes 1–4 contains 24, 48, 72, 96 h by methanol induction. Samples of culture supernatants that were 15-fold concentrated with TCA.

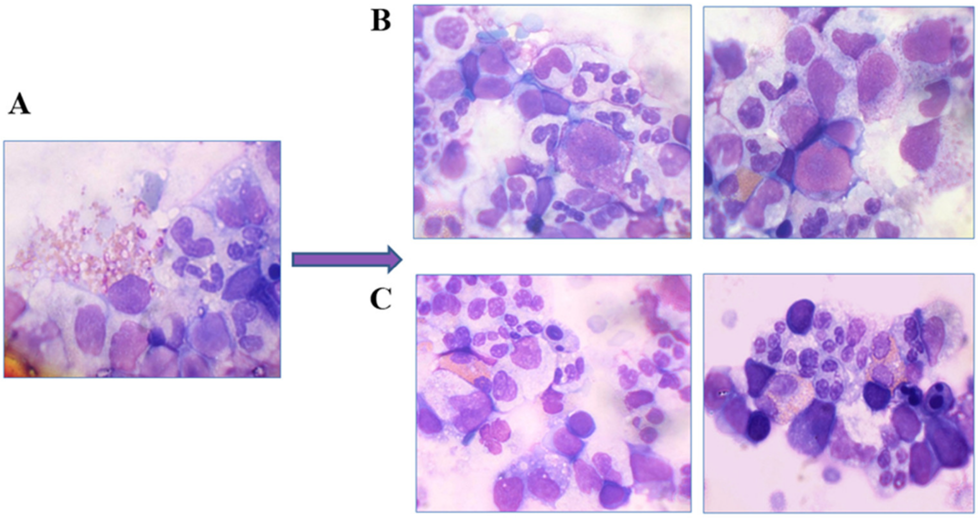

Figure S3. Human BMCs treated with ryGM-CSF–ApoA-I (B) and ryGM-CSF (C) versus control (A) for 48 h of incubation. The control is observed: Immature hyposegmented neutrophils, increased apoptosis, phagocytosis, increased number of monocytes. Samples treated with ryGM-CSF–ApoA-I - mature neutrophils, viable intact stroma, blast cells. Samples treated with ryGM-CSF - mature neutrophils with less condensed chromatin, monocytes, macrophages, eosinophils and erythroid blasts, apoptosis.
